# Identification of Hub Genes and Key Pathways Associated with Two Subtypes of Diffuse Large B-Cell Lymphoma Based on Gene Expression Profiling via Integrated Bioinformatics

**DOI:** 10.1155/2018/3574534

**Published:** 2018-05-24

**Authors:** Zijian Liu, Jingshu Meng, Xiaoqian Li, Fang Zhu, Tao Liu, Gang Wu, Liling Zhang

**Affiliations:** Cancer Center, Union Hospital, Tongji Medical College, Huazhong University of Science and Technology, Wuhan, China

## Abstract

There is a significant difference in prognosis between the germinal center B-cell (GCB) and activated B-cell (ABC) subtypes of diffuse large B-cell lymphoma (DLBCL). However, the signaling pathways and driver genes involved in these disparate subtypes are ambiguous. This study integrated three cohort profile datasets, including 250 GCB samples and 250 ABC samples, to elucidate potential candidate hub genes and key pathways involved in these two subtypes. Differentially expressed genes (DEGs) were identified. After Gene Ontology functional enrichment analysis of the DEGs, protein-protein interaction (PPI) network and sub-PPI network analyses were conducted using the STRING database and Cytoscape software. Subsequently, the Oncomine database and the cBioportal online tool were employed to verify the alterations and differential expression of the 8 hub genes (MME, CD44, IRF4, STAT3, IL2RA, ETV6, CCND2, and CFLAR). Gene set enrichment analysis was also employed to identify the intersection of the key pathways (JAK-STAT, FOXO, and NF-*κ*B pathways) validated in the above analyses. These hub genes and key pathways could improve our understanding of the process of tumorigenesis and the underlying molecular events and may be therapeutic targets for the precise treatment of these two subtypes with different prognoses.

## 1. Introduction

Diffuse large B-cell lymphoma (DLBCL) is the most common type of adult non-Hodgkin lymphoma (NHL) in Western countries, accounting for 25-30% of all cases of NHL; in 2015, it was estimated that approximately 24,000 new cases/year are diagnosed in the United States [1, 2]. Recent gene expression microarray analyses of DLBCL have revealed significant heterogeneity within this diagnosis, including the germinal center B-cell (GCB) and activated B-cell (ABC) DLBCL subtypes, which are derived from B cells at different stages of differentiation. GCB DLBCL appears to arise from GCBs, whereas ABC DLBCL likely arises from post-GCBs that are blocked during plasmacytic differentiation [3, 4]. The LNH-98.5 trial confirmed that R-CHOP improves patient outcomes in elderly DLBCL patients and that the beneficial effects are sustained over a long follow-up period compared with the CHOP treatment regimen [5]. Analysis of molecular subtypes and outcomes following upfront CHOP treatment showed a statistically significant difference in 5-year OS between the DLBCL subtypes: 59% for GCB DLBCL and 31% for ABC DLBCL, independent of IPI (International Prognostic Index) risk group [6]. Another analysis performed on 233 biopsies obtained from patients treated with R-CHOP indicated that patients with GCB DLBCL exhibited more favorable survival outcomes than those with ABC DLBCL, with 3-year OS rates of 84% and 56%, respectively (P<0.001); as expected, the OS of both GCB and ABC DLBCL patients recorded in this study was better than that in the prerituximab era [4]. Following the same therapeutic method, the significantly different prognostic characteristics of these two DLBCL subtypes revealed differences in the molecular pathogenesis of the two subgroups leading to their disparate survival rates and prognoses. Thus, elucidating the causes and the underlying molecular mechanisms of these DLBCL subtypes and identifying molecular biomarkers for diagnosis, prevention, and personalized therapy are critically important.

Gene chip analysis, or gene profiling, is a gene detection technique that has been used for more than ten years. Gene chips can quickly generate expression information for all of the genes in a sample at a given time point and are particularly suitable for differentially expressed genes (DEGs) screening [7]. Integration and reanalysis of data deposited and stored in public databases provide valuable clues for new research, and many microarray data profiling studies have been performed on DLBCL in recent years. However, these results were generated from a single cohort study, and there are limitations due to tissue or sample heterogeneity in independent studies. Nevertheless, the combination of integrated bioinformatics methods with expression profiling could be revolutionary and overcome these shortcomings.

The NCBI-Gene Expression Omnibus Database (NCBI-GEO) has facilitated such analyses by furnishing several microarray datasets, including the GSE53786, GSE56315, and GSE31312 datasets, including a total of 250 GCB samples and 250 ABC samples. We used the R package to analyze aberrantly expressed genes, performed Gene Ontology (GO) and pathway enrichment analysis to screen differentially expressed genes (DEGs) at the Database for Annotation, Visualization and Integrated Discovery (DAVID) website, integrated the DEGs into a protein-protein interaction (PPI) network, and performed modular analysis using the Search Tool for Retrieval of Interacting Genes/Proteins (STRING) database and Cytoscape software to identify hub genes. Then, the Oncomine database, the cBioportal online tool, and gene set enrichment analysis (GSEA) were utilized to validate the reliability and authenticity of the hub genes in the two different DLBCL subtypes. The identification of significant DEGs, enrichment of their biological functions and key pathways, and visualization of the network of DEGs and hub genes will provide more accurate and reliable biomarkers and therapeutic targets for early diagnosis, individualized prevention measures, and improvement of therapeutic efficacy.

## 2. Materials and Methods

### 2.1. Microarray Data Information and DEG Identification

NCBI-GEO is a free database of microarray/gene profiles and next-generation sequencing data, from which three DLBCL GCB- and ABC-subtype gene expression profiles (GSE53786, GSE56315, and GSE31312) were obtained. These datasets were based on the GPL570 platform ([HG-U133_Plus_2] Affymetrix Human Genome U133 Plus 2.0 Array, Santa Clara, CA, USA). A total of 324 DLBCL samples were included in GSE31312, comprising 160 samples from the GCB subtype and 164 samples from the ABC subtype (submission date: August 10, 2011) [8]. GSE56315 (submission date: Mar 27, 2014) and GSE53786 (submission date: January 02, 2014) included 44 GCB- and 46 ABC-subtype samples and 46 GCB- and 40 ABC-subtype samples, respectively [9, 10]. We chose these three datasets for our integrated analysis in this study because these datasets, which include both the GCB and ABC subtypes, were generated from the same sequencing platform. Thus, we could integrate these datasets in our subsequent processing steps.

The Robust Multiarray Average (RMA) algorithm in the Bioconductor package (http://www.bioconductor.org/) was applied to the raw data for high-throughput functional genomic expression, including background correction, quartile normalization, and probe summarization. The linear models for microarray data (LIMMA, http://www.bioconductor.org/packages/release/bioc/html/limma.html) package in Bioconductor were utilized to mine statistically significant DEGs based on the difference in their expression values between samples of the GCB and ABC subtypes. We defined the corresponding* p* values for genes after the T-test as the adjusted* p* value, and an adjusted* p* value < 0.05 and a |log2FC(fold change)|≥1 were defined as the cut-off criteria.

### 2.2. GO and Pathway Enrichment Analysis

The functions and pathway enrichment of candidate DEGs were analyzed using DAVID (http://david.abcc.ncifcrf.gov/) [11], which is a gene functional classification implement that accommodates a set of functional annotation tools for investigators to analyze the biological roles of genes and be used to perform GO and KEGG (Kyoto Encyclopedia of Genes and Genomes) pathway enrichment analyses of DEGs. A count≥2 and EASE >0.1 were considered as the cut-off criteria.

### 2.3. Integration of the PPI Network, Molecular Analysis, and Identification of Significant Candidate Genes and Pathways

Using the online STRING (http://string-db.org/) database [12], which is a biological database and web resource for known and predicted PPIs, we developed a network of DEG-encoded proteins and PPIs. Cytoscape software [13] was applied to visualize the protein interaction relationship network and analyze hub proteins, which are important nodes with many interaction partners. We utilized the CytoHubba application in Cytoscape, employing five calculation methods: Degree, EPC, EcCentricity, MCC, and MNC. The intersecting genes derived using these five algorithms encode core proteins and may represent key candidate genes with important biological regulatory functions. ClusterONE, an application in Cytoscape, was utilized to identify the crucial modules for further analysis. ClueGO and CluePedia were also employed to draw KEGG pathways for visualization purposes.

### 2.4. Validation of the Aberrant Expression and Clinical Value of Hub Genes

To verify the genetic alterations associated with these hub genes, including amplifications, deletions, or point mutations, cBioPortal (available online: http://www.cbioportal.org/), a tool developed by the Computation Biology Center at Sloan Kettering, was used to summarize possible transcriptional changes, mutual expression tendencies, and overall survival through Kaplan-Meier analysis, and the results were presented as OncoPrint and mutual exclusivity or cooccurrence data. The clinical value of the genetic alterations was also evaluated. Furthermore, datasets from the Oncomine database (http://www.oncomine.org) were extracted to validate the highly differentiated hub genes between the GCB and ABC subtypes.

### 2.5. GSEA

GSEA is a method employed to analyze and interpret microarray data using biological technology and has been previously described [14]. Preconditioned GEO data are analyzed based on differential enrichment in a predefined coexpression or biological pathway (gene set) from a previous experiment. If the majority of a gene set exhibits high expression accompanied by a high risk score, the gene set will present a positive enrichment score and will be referred to as ‘enriched'[15]. GSEA was downloaded from GSEA HOME (http://software.broadinstitute.org/gsea/index.jsp) and run in a Java environment. Significant gene sets with an FDR <25% and a nominal* p* value < 0.05 were identified.

## 3. Results

### 3.1. Identification of DEGs between the GCB and ABC Subtypes

**Figure 1:**
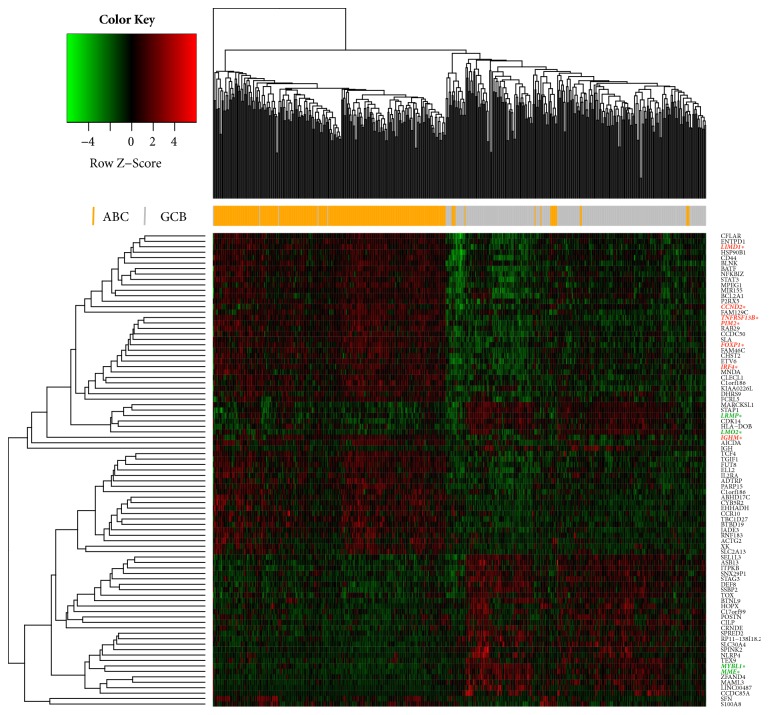
Heatmap of the DEGs. Each raw read represents a single gene, and each column represents a tissue sample. The gradual color change from green to red represents the shift from ABC-upregulated to ABC-downregulated genes. This analysis revealed 87 DEGs that were significantly different between the ABC and GCB tissues. ^*∗*^COO common markers are in red (ABC-markers) or green (GCB-markers).

**Figure 2:**
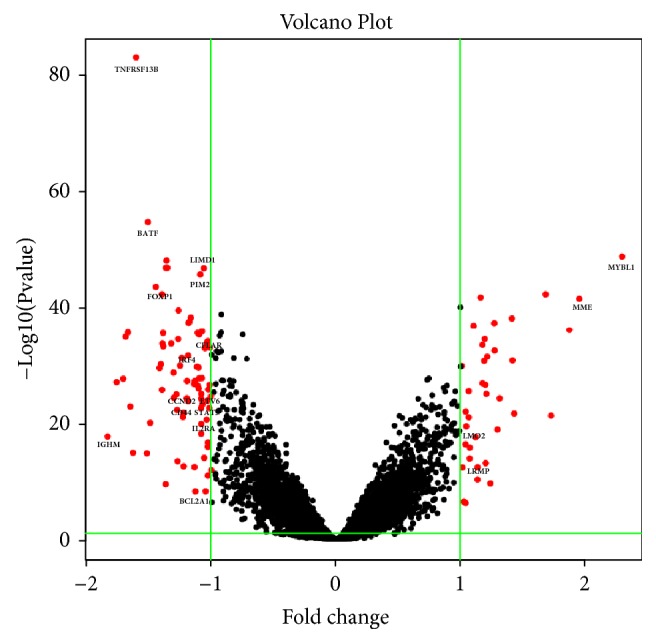
Volcano plot showing the aberrantly expressed genes between the ABC and GCB samples. Red dots indicate the genes showing an expression difference with a |log2 FC|>1 and* p* < 0.05, while the black dots fail to meet these criteria. The ABC-upregulated DEGs are displayed on the left of the plot, and the downregulated DEGs are on the opposite side. Hub genes and some genes with highest adjust* p* value and |log2 FC| value were marked in the figure. The x-axis represents the log2 FC score, and the y-axis shows the –log10 value (*P* value). This volcano plot was generated using R language (R 3.4.0).

After removing batch differences and performing data normalization, a total of 33 ABC-downregulated DEGs and 54 ABC-upregulated DEGs were obtained based on the cut-off criteria (*p* < 0.05 and |log2 FC|>1) (Table 1). The hierarchical cluster analysis of the posttreatment data demonstrated that the DEGs accurately distinguished the ABC samples from the GCB samples (Figure 1). The heatmap.2 package in Bioconductor was utilized to visualize the cluster analysis, with parameters including Euclidean distance and the average algorithm. Using the calculated criteria, all the aberrantly expressed genes with log2 FC scores and –log10* p* values were used to generate a volcano plot in R language, which is a visual tool for showing DEGs among overall gene expression levels (Figure 2).

### 3.2. Enrichment Analyses of the DEGs

**Figure 3:**
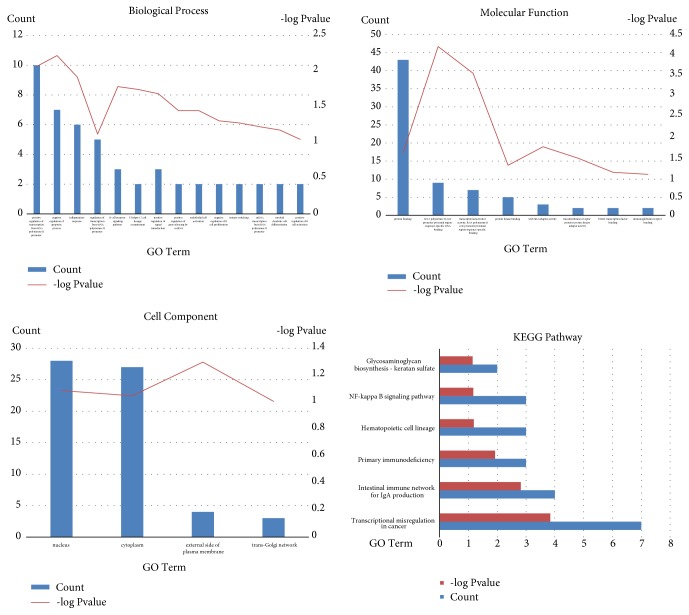
GO analysis and significantly enriched GO terms for DEGs in DLBCL.GO analysis classified the DEGs into three groups (molecular function, biological process, and cellular component). Significantly enriched GO terms and KEGG pathways for DEGs in DLBCL based on their functions.

The functions and pathway enrichment of the candidate DEGs were evaluated at the DAVID website. GO analysis further classified the DEGs into the following three functional groups: (1) cellular components, (2) molecular functions, and (3) biological processes (Figure S1). As shown in Figure 3 and Table 2, the DEGs were mainly enriched in transcription, apoptotic processes, inflammatory responses, and B-cell or T-cell signaling pathways in the biological process group. In the cellular component group, aberrantly expressed genes were mainly enriched in the nucleus and cytoplasm. In the molecular function group, transcriptional activity and protein binding were the main areas of enrichment. Most of the enriched genes came from the ABC-upregulated group; the ABC-downregulated group made a lesser contribution, with the majority of these genes displaying no significant enrichment. These results showed that most of the DEGs were significantly enriched in transcription processes, B- or T-cell signaling pathways, cell components, and various types of binding. According to the KEGG (Kyoto Encyclopedia of Genes and Genomes) pathway enrichment analysis, the DEGs were mainly enriched in transcriptional misregulation in cancer, the intestinal immune network for IgA production, the hematopoietic cell lineage, and the NF-*κ*B signaling pathway (Figure 3 and Table 3). Only a small fraction of the genes extracted from the ABC-downregulated genes were enriched in any pathway; these genes included LMO2, CDK14, MME, and HLA-DOB.

### 3.3. Identification of Hub Genes and Pathways through DEG PPI Network Analysis

**Figure 4:**
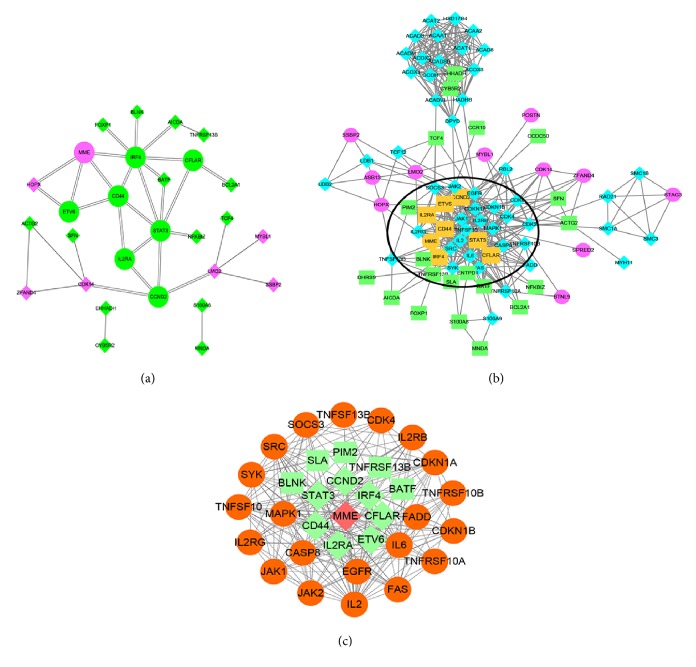
DEG PPI network complex and modular analysis. (a) Using the STRING online database, 28 of 87 DEGs (21 ABC-upregulated genes in green, 7 downregulated genes in purple; hub genes are indicated with round shapes) were filtered into the DEG PPI network complex. (b) According to the above method, other networks with no more than 50 interactors were also analyzed. The hub genes are shown in orange, and the other genes linked to the DEGs are shown in blue. The highlighted circled areas are the most significant modules. (c) This module consists of 34 nodes and 222 edges (ABC-upregulated genes in green; downregulated gene in red; hub genes are indicated with diamond shapes and DEGs are indicated with rectangle shapes; and other linked genes are in orange and with round shapes), which are mainly associated with the regulation of biological processes, cellular components, and protein binding.

**Figure 5:**
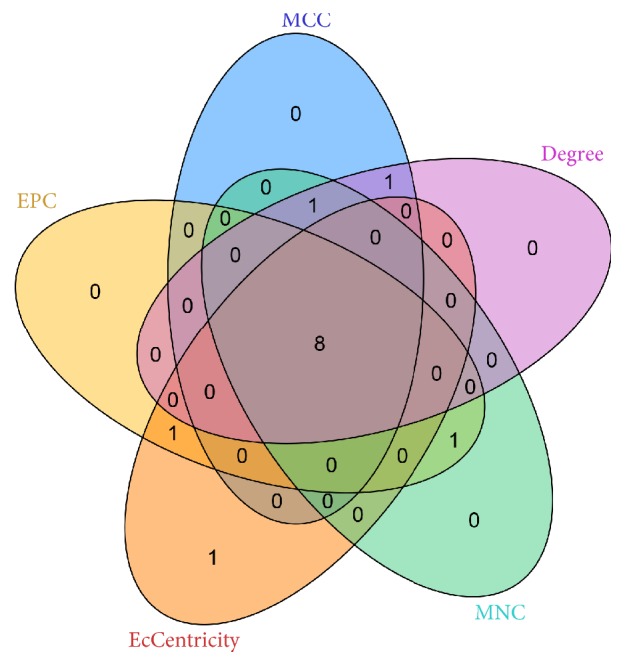
Employing an online resource, we used five intersecting algorithms to generate a Venn plot to identify significant hub genes. Areas with different colors correspond to different algorithms. The cross areas indicate the commonly accumulated DEGs. The elements in concurrent areas are the 8 hub genes (MME, CD44, IRF4, STAT3, IL2RA, ETV6, CCND2, and CFLAR).

**Figure 6:**
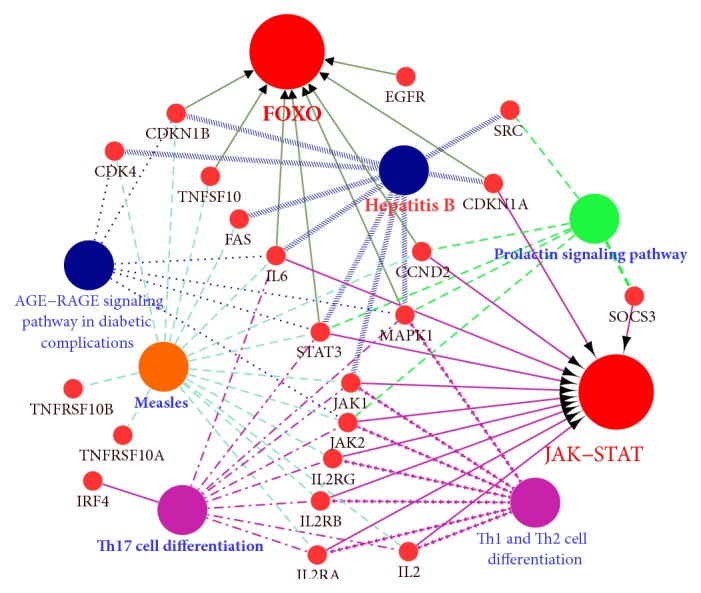
The KEGG pathways for the module were visualized in Cytoscape with ClueGo and CluePedia. The larger nodes represent the enriched pathways, while the smaller nodes represent the genes in the significant module that interact with these enriched pathways.

A PPI network containing 28 nodes and 34 interactions was constructed using the STRING online database, with parameters including a minimum required interaction score > 0.4 (medium confidence) and only query proteins being displayed; thus, 28 of the 87 DEGs were included in the DEG PPI network (Figure 4(a)). After importing the data into Cytoscape and running the CytoHubba application, the top 10 genes evaluated by the five calculation methods (Degree, EPC, EcCentricity, MCC, and MNC) were listed (Table 4). Furthermore, using an online website (http://www.ehbio.com/ImageGP/index.php/Home/Index/VennDiagram.html), we observed the intersections of these five algorithms and generated a Venn plot to identify significant hub genes (Figure 5). The 8 most significant genes were MME, CD44, interferon regulatory factor 4 (IRF4), signal transducer and activator of transcription 3 (STAT3), interleukin 2 receptor subunit alpha (IL2RA), ETV6, cyclin D2 (CCND2), and CASP8 and FADD like apoptosis regulator (CFLAR), all of which interacted with one another and were employed to perform GO analysis with the STRING database (Table 5). These hub genes were chiefly enriched in cytokine signaling or biological processes and the JAK-STAT and FOXO signaling pathways. In the case of increased interactions between our DEGs and other proteins, other networks with no more than 50 interactors were also analyzed (Figure 4(b)), and one important module was constructed using ClusterONE for further investigation (Figure 4(c)). Strikingly, all the DEGs in this module were upregulated genes, and each hub gene was embodied in this module. GO enrichment analysis revealed that this module consisted of 34 nodes and 222 edges, which were mainly associated with the regulation of biological processes, cellular components, and protein binding (Table 6). In the KEGG pathway analysis, the highest-ranking pathways included cytokine-cytokine receptor interactions and the JAK-STAT and FOXO signaling pathways, which compared well with the hub gene pathways (Figure 6).

### 3.4. Validation of Hub Genes in the Oncomine Database

The Oncomine database was utilized to explore the expression of the hub genes between the ABC and GCB subtypes of DLBCL. We used search terms and isolated datasets representing lymphoma histology analysis and GCB versus ABC. An analysis of a representative dataset (Salaverria lymphoma) revealed that MME expression levels were significantly higher in the GCB subtype than those in the ABC subtype and that IL2RA, ETV6, and CCND2 expression levels were higher in the ABC subtype than those in the GCB subtype. Another lymphoma dataset (Compagno Lymphoma Statistics) indicated that the expression levels of CD44 and STAT3 were higher in ABC than those in GCB. The expression levels of both IRF4 and CFLAR were higher in ABC than those in GCB (investigated in the Lenz Lymphoma Statistics and Zhang Lymphoma Statistics datasets, respectively) (Figure S2). For further validation, we identified 7 datasets that included ABC and GBC gene expression to perform a comparative analysis to observe the expression levels of the 8 hub genes in each dataset. In the comparative analysis, almost every pair of datasets indicated that there was a significant difference between the ABC and GCB groups. Thus, all the hub genes showed significantly higher expression levels in the ABC subtype than those in the GCB subtype, except for MME, which showed lower expression in the ABC subtype.

### 3.5. Analysis of the Potential Molecular Mechanisms of Hub Genes and Their Prognostic Influence in cBioPortal

**Figure 7:**
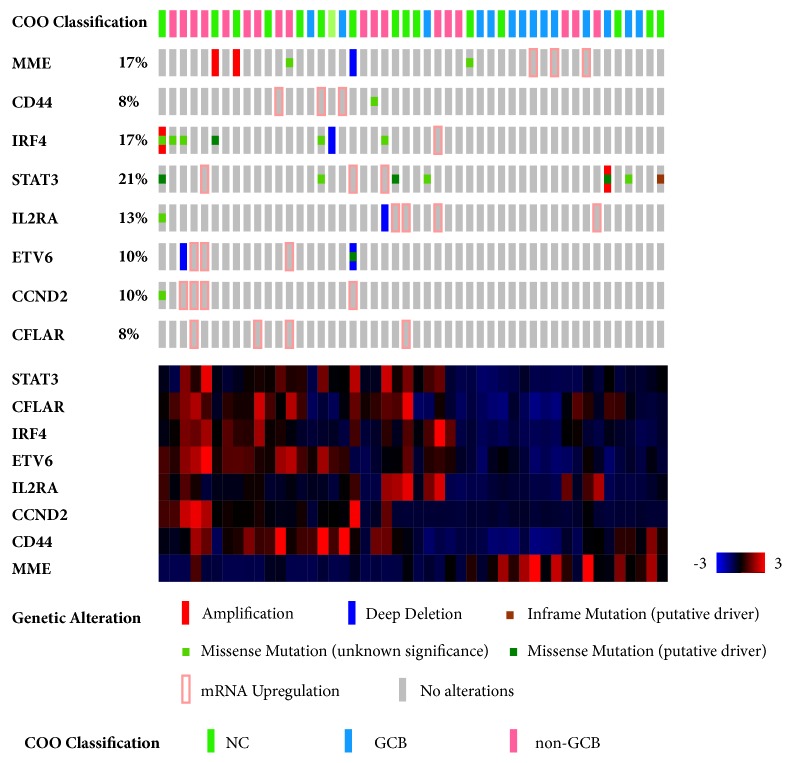
OncoPrint in cBioportal revealed that 58% (28/48) of cases displayed genetic alterations, including amplification, severe depletion, mRNA upregulation, and various mutations. The cluster heatmap indicated that cases with higher MME expression exhibited a tendency to display lower expression of the seven other hub genes, and vice versa.

To investigate the clinical significance of the hub genes, the genetic changes in the hub genes were verified by interrogating cBioportal, an online tool that can analyze datasets extracted from TCGA database. OncoPrint from cBioportal revealed that 58% (28/48) of cases exhibited genetic alterations, including amplification, severe depletion, mRNA upregulation, and various mutations. The cluster heatmap indicated that one case with higher MME expression potentially displayed a tendency toward lower expression of seven other hub genes (Figure 7). cBioportal also provided the probable mutual exclusivity or cooccurrence of these hub genes. As shown in Supplemental Table 1, there was a significant tendency towards cooccurrence between ETV6 and CCND2 and between CFLAR, IRF4, and IL2RA in DLBCL. More interestingly, the cases with genetic alterations in ABC-upregulated hub genes displayed poorer survival compared with the cases without alterations, while the same cases showing changes in MME showed better survival (Figure S3). The overall survival of each ABC-upregulated gene was analyzed, and statistical significance was only found for IRF4 (Figure S3). One limitation of the cBioportal analysis was that none of the cases were divided into ABC and GCB groups. However, genetic alterations and up- or downregulation of these hub genes could be demonstrated.

### 3.6. GSEA

**Figure 8:**
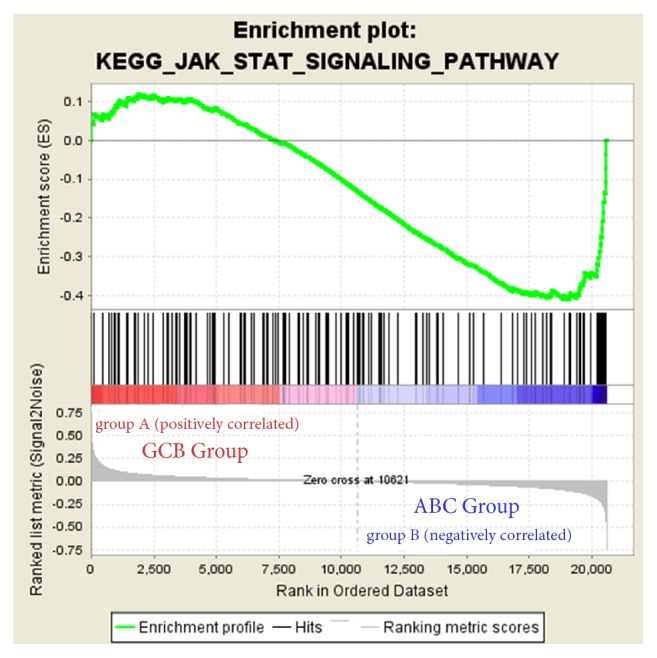
GSEA analysis revealed that ABC-upregulated genes were enriched in the JAK-STAT signaling pathway (ES = -0.41, NOM* p* value = 0.006, FDR = 0.208, and FWER* p* value = 0.775). A positive enrichment score (ES) indicated correlation with the first group (GCB) and a negative ES indicated correlation with the second group (ABC).

Here, we employed GSEA as a conventional approach for identifying pathways related to the differences in the ABC and GCB subtypes. The results revealed no significant gene sets at an FDR<25% in the GCB group, but 42 gene sets were identified in the ABC group. Among the significant gene sets in the ABC group, the JAK-STAT signaling pathway comprised the most genes, with 151 genes involved in this pathway (ES=-0.41, NOM p-val=0.006, FDR=0.208 and FWER p value=0.775) (Figure 8). Some important pathways, such as the TGF-*β*, WNT, and GnRH signaling pathways, failed to meet the screening criteria, such as the FDR score requirement. Only the JAK-STAT signaling pathway overlapped between the GSEA and the former analysis with a significant difference.

## 4. Discussion

In the present study, an integrated bioinformatics analysis was performed to explore potential crucial genes and key pathways associated with the GCB and ABC subtypes in DLBCL. In this study, three cohort profile datasets from different groups were integrated to thoroughly examine the information they contained using bioinformatics methods, and 87 commonly altered DEGs were identified (33 downregulated and 54 upregulated). Some genes are commonly recognized as GCB or ABC-specific markers. For instance, the expressions of MYBL1, MME, LRMP, and LMO2 are related to GCB subtypes, and the expression of FOXP1, IRF4, IGHM, TNGRSF13B, PIM2, CCND2, and LIMD1 can be commonly biomarkers of ABC subtype [3, 4, 16, 17]. Their expression level can be utilized as differentiation and prognostic indicators. The up- and downregulated DEGs were classified into three groups (biological processes, molecular functions, and cellular components) according to GO terms, and KEGG pathway enrichment analysis was conducted using the DAVID website. The upregulated DEGs were mainly involved in functions related to the transcription process, B- or T-cell signaling pathways, cellular components, and various types of binding, while the downregulated DEGs were not found to be significantly enriched in this study. The most enriched pathways pertained to transcriptional misregulation in cancer, the intestinal immune network for IgA production, the hematopoietic cell lineage, and the NF-*κ*B signaling pathway. In the PPI network module, downregulated MME and upregulated CD44, IRF4, STAT3, IL2RA, ETV6, CCND2, and CFLAR were identified as the crucial hub genes based on five hub gene calculation algorithms. These genes were mainly enriched in cytokine signaling or biological processes and the JAK-STAT and FOXO signaling pathways. For further investigation, we expanded the network of interactions to show other genes linked with the DEGs, to validate the GO and pathway enrichment of these DEGs. Finally, the most significant module, containing all the hub genes, was filtered, and most of the corresponding genes were associated with the regulation of biological processes, protein binding, and the JAK-STAT and FOXO signaling pathways, similar to the other analyses.

DLBCL is a heterogeneous disease from several points of view, including its morphology, immunophenotype, and molecular features, which is also reflected by its pathogenetic mechanisms [1, 16]. In a previous study, it was confirmed that the expression of the BCL2 protein and the ABC subtype significantly increase with age, which are both adverse prognostic features [17–19]. Another study showed that MYC protein expression is associated with age and that MYC/BCL2 and MYC/BCL6 double expression might be enriched in older age groups, although this remains to be validated in future studies [20]. Moreover, while Bcl-6 protein expression is a marker of germinal center derivation, it has also been identified as one of the strongest predictors of DLBCL outcomes with a favorable prognosis [21]. The important role of changes in the NF-*κ*B family is emphasized by evidence showing that NF-*κ*B induces resistance to apoptosis [22]. The aims of this study were to analyze new markers in DLBCL biology and contribute to the development of potential new treatment options.

Using integrated bioinformatics, we identified 8 hub genes, among which only one was upregulated in the GCB subtype, while the others were upregulated in the ABC subtype. MME, the only hub gene upregulated in the GCB subtype, is a membrane metalloendopeptidase that is involved in cellular responses to cytokine stimuli and hematopoietic cell lineages. IRF4 plays an important role in the cytokine-mediated signaling pathway, is normally expressed during lymphocyte activation, and has been shown to be an important component of proliferative stimulation [23]. There is a great deal of overlap between CCND2, G1/S-specific cyclin D2, and STAT3, including the prolactin and FOXO signaling pathways, which are inactivated by major oncogenic signals such as the PI3K and MAPK pathways, and their expression is also repressed by microRNAs in multiple cancer types [24]. Furthermore, the JAK-STAT signaling pathway has been demonstrated to be linked to the transcriptional regulation of genes related to development and innate immunity and to an intracellular signaling pathway regulating cytokine signaling [25–28]. Additionally, IL2RA (CD25), which is vital in leukemogenesis and may stabilize oncogenic tyrosine kinase signaling by mediating negative feedback pathways in leukemic cells, is involved in the JAK-STAT signaling pathway with CCND2 and STAT3 [29–31]. This interconnection may therefore make IL2RA an attractive target for further studies on the DLBCL ABC subtype. Interestingly, these three hub genes are also enriched in measles; thus, it is important that further research be conducted in this arena. In a previous study, CD44 expression was associated with the non-GC phenotype and should therefore be studied as a promising candidate for biological marker screening in systemic DLBCL patients [32]. The high expression of CFLAR in the ABC subtype may facilitate cell proliferation and reduce TRAIL-induced apoptosis [33]; this previous hypothesis was verified in our analysis by the enrichment of CFLAR in the regulation of satellite cell proliferation. Although the translocations or deletions of ETV6 gene are reported in a variety of hematologic neoplasms, including acute myeloid and lymphoblastic leukemia, myelodysplastic syndrome, and myeloproliferative disorders, it is rarely reported in DLBCL [34, 35]. A mutational analysis of primary central nervous system lymphoma also revealed that ETV6 might be an underlying target for this kind of lymphoma treatment [36]. In recent study [37], ETV6 and other 149 genes are identified as driver genes of DLBCL. However, the functional role and transcriptional regulation pathway of ETV6 in DLBCL are still deficient, which can be a novel biomarker in basic research later.

In a previous study, the most important hallmark of the ABC subtype was found to be upregulation of BCR signaling by the NF-*κ*B proliferative pathway [38, 39]. In this analysis, CFLAR, BLNK, and BCL2A1 were enriched in this signaling pathway; thus, these three genes might be investigated as new therapeutic targets in future research. BLNK is a B-cell linker that bridges B-cell receptor-associated kinases with a multitude of signaling pathways and may regulate the biological outcomes of B-cell function and development [40]. An intestinal immune network for IgA production might provide new markers in enteric DLBCL, such as CCR10, TNFRSF13B, AICDA, and HLA-DOB, as well as genes involved in transcriptional misregulation in cancer (NFKBIZ, FUT8, LMO2, CCND2, BCL2A1, ETV6, and CDK14). In the present study, we further interrogated other networks with no more than 50 interactors to obtain more useful information. It was found that a significant module or cluster consisting of 34 genes, including all the hub genes and many ABC-upregulated genes, was enriched in biological processes, protein binding, and pathways including cytokine-cytokine receptor interaction and the JAK-STAT and FOXO signaling pathways. Therefore, these GO functions and KEGG pathways will be highly significant when defining our next steps. In addition to the enrichment of the JAK-STAT and FOXO signaling pathways in the module, Th17 cell differentiation, Th1 and Th2 cell differentiation, and hepatitis B and measles were also enriched in this module. Previous studies have demonstrated that NHL is closely related to Th17 cells and associated cytokines, whose levels are significantly lower in peripheral blood from DLBCL patients but are increased in relapse patients [41, 42]. Moreover, changes in the Th1/Th2 ratio are usually highlighted in NHL [43], and newly diagnosed aggressive B-cell NHL is associated with the Th1/Th2 balance [44]. Hence, the genes involved in these pathways might provide a new direction of research on the original basis.

Considering our results, we interrogated the Oncomine database to perform a comparative analysis with 7 datasets, to verify the differential expression of the hub genes. The results were strongly in line with our preceding expectations, showing that MME was upregulated in the GCB subtype, while the other genes were upregulated in the ABC subtype in the 7 datasets established through our predecessors' efforts. These genes were also imported into the cBioportal online tool to analyze their expression in TCGA database for clinical research, which focuses on genetic alterations and overall survival curves. It was revealed that 58% (28/48) of cases displayed genetic alterations, including amplification, severe depletion, mRNA upregulation, and various mutations. For the overall survival of cases with alterations in these mentioned genes, only IRF4 showed statistical significance; nevertheless, this result remains exciting in relation to the assessment of the prognosis of patients with changes in these genes. However, it does not mean that other analyses are not useful for prognostic evaluation; they only require more thoughtful study. More importantly, cBioportal provided the probable mutual exclusivity or cooccurrence of these hub genes and showed that ETV6 exhibited a significant cooccurrence tendency with CCND2 and CFLAR, similar to IRF4 and IL2RA. CFLAR is enriched for the same GO terms and pathways, although ETV6 does not show the same enrichment as CCND2 and CFLAR. This means that ETV6 could participate in the same biological processes and pathways as CCND2 and CFLAR. However, ETV6 has been highly discussed in relation to adult acute leukemia [45], which is associated with juvenile cells, while DLBCL is mainly linked to mature cells. Thus, these results deserve further study in our follow-up work. Comparison of the results of GSEA and the previous pathway analysis showed that the JAK-STAT signaling pathway was our key pathway and that it is important to determine how the genes related to the network regulate this pathway in DLBCL.

Consistent with our results, previous studies have also identified DEGs in DLBCL. For example, T. Dandekar's group analyzed patients classified into GCB and ABC groups to identify molecular markers using tedious methods. Some of the genes identified by those authors showed involvement in our analysis as well, such as MME, CCND2, and IRF4. In contrast to our predecessor's work, we accumulated three large datasets containing 500 samples and utilized GO term analysis and Cytoscape software to obtain more reasonable results and visualize the results. Databases such as Oncomine and cBioportal were interrogated to verify the DEGs. Other studies based on one of the databases (GSE31312) selected in the present study have focused more on therapeutic and prognostic value, based on R-CHOP or the NF-*κ*B signaling pathway.

DLBCL is a group of histologically and molecularly heterogeneous diseases characterized by different sets of genetic and epigenetic alterations involved in multiple functional signaling pathways that are modulated by genetic events, leading the alterations in transcriptional and/or translational levels. Thus, factors such as gene mutations, hypo- or hypermethylation, microRNAs, and lncRNAs should be considered as well. This is one deficiency of this study in drawing the specific conclusion that it is changes in these mRNAs that cause the transformation of these signaling pathways. Another limitation of this study is the lack of validation experiments, which will be conducted in our future work.

## 5. Conclusions

Taken together, the results obtained using multiple cohort profile datasets and integrated analysis led to the identification of 87 DEG candidate genes and 28 gene nodes in a PPI network and, thus, the elucidation of the 8 most connected hub genes (MME, CD44, IRF4, STAT3, IL2RA, ETV6, CCND2, and CFLAR). These genes were significantly enriched in several different biological processes and signaling pathways, mainly associated with transcription and cytokine-mediated processes, apoptosis, the hematopoietic cell lineage, and certain vital pathways, including the JAK-STAT, FOXO, and NF-*κ*B signaling pathways. These findings could significantly improve our understanding of the differences in the GCB and ABC subtypes as well as the process of tumorigenesis and underlying molecular events. These candidate hub genes and key pathways could be the therapeutic targets for the precise treatment of these two subtypes with different prognoses.

## Figures and Tables

**Table 1:** Eighty-seven differentially expressed genes (DEGs), 54 upregulated genes and 33 downregulated genes, were identified from 3 profile datasets in the ABC samples compared with those in the GCB samples. The DEGs are listed from the largest to the smallest fold change in the table.^*∗*^ GCB common markers. ^*∗∗*^ ABC common markers.

**DEGs**	**Gene Names**
**ABC Down-regulated**	MYBL1^*∗*^, MME^*∗*^, LINC00487, C17orf99, STAG3, BTNL9, STAP1, CCDC85A, RP11-138I18.2, SSBP2, IGHE, SEL1L3, SNX29P1, CILP, SPINK2, MARCKSL1, ITPKB, MAML3, SPRED2, ASB13, HOPX, CRNDE, LRMP^*∗*^, ZFAND4, TEX9, NLRP4, DEF8, LMO2^*∗*^, SLC30A4, POSTN, HLA-DOB, TOX, CDK14

**ABC Up-regulated**	IGHM^*∗∗*^, XK, KIAA0226L, MIR155 (MIR155HG), CLECL1, AICDA, TNFRSF13B^*∗∗*^, BATF, FAM129C, FOXP1^*∗∗*^, C1orf186 (LOC101929219), P2RX5, FUT8, IRF4^*∗∗*^, CYB5R2, SFN, TBC1D27, JADE3, PARP15, ADTRP, MNDA, FCRL5, FAM46C, MPEG1, BLNK, CD44, CCND2^*∗∗*^, BTBD19, EHHADH,RAB29, CCDC50, DHRS9, CHST2, S100A8, SLA, CFLAR, PIM2^*∗∗*^, STAT3, ELL2, IL2RA, ETV6, NFKBIZ, ENTPD1, HSP90B1 (MIR3652), LIMD1^*∗∗*^, ACTG2, TGIF1, SLC2A13, ABHD17C, RNF183, TCF4, CCR10, BCL2A1

**Table 2:** Analysis of significant enrichment of DEGs in DLBCL.

**Term**	**Description**	**Count**	**P Value**	**Genes**
**Biological process**				

GO:0045944	positive regulation of transcription from RNA polymerase II promoter	10	0.008568	BATF, SSBP2, LMO2, MAML3, IRF4, MYBL1, ETV6, TCF4, FOXP1, STAT3
GO:0043066	negative regulation of apoptotic process	7	0.006042	CFLAR, HSP90B1, CD44, CCND2, BCL2A1, PIM2, STAT3
GO:0006954	inflammatory response	6	0.012073	NFKBIZ, IL2RA, NLRP4, S100A8, CHST2, BLNK
GO:0050853	B cell receptor signaling pathway	3	0.016456	IGHE, MNDA, IGHM
GO:0009967	positive regulation of signal transduction	3	0.020706	STAP1, BLNK, SLA

**Cell component**				

GO:0005634	nucleus	28	0.081986	CYB5R2, S100A8, LMO2, ITPKB, MYBL1, SFN, BATF, STAG3, LIMD1…
GO:0005737	cytoplasm	27	0.089649	FUT8, MARCKSL1, EHHADH, MME, POSTN, SFN, BATF, ACTG2, CD44…

**Molecular function**				

GO:0005515	protein binding	43	0.027431	CYB5R2, S100A8, LMO2, MARCKSL1, EHHADH, SPINK2, XK…
GO:0000978	RNA polymerase II core promoter proximal region sequence-specific DNA binding	9	6.28E-05	BATF, SSBP2, TGIF1, IRF4, MYBL1, ETV6, TCF4, FOXP1, STAT3
GO:0001077	transcriptional activator activity, RNA polymerase II core promoter proximal region sequence-specific binding	7	2.91E-04	BATF, SSBP2, IRF4, MYBL1, ETV6, TCF4, STAT3
GO:0005515	protein kinase binding	5	0.056821	STAP1, CCND2, SPRED2, SFN, STAT3
GO:0005070	SH3/SH2 adaptor activity	3	0.019671	STAP1, BLNK, SLA

**Table 3:** Significantly enriched pathway terms for DEGs in DLBCL. Functional and signaling pathway enrichment analyses of the DEGs were conducted using the DAVID website.

Term	KEGG Pathway	Count	P Value	Genes
hsa05202	Transcriptional misregulation in cancer	7	0.00014	NFKBIZ, FUT8, LMO2, CCND2, BCL2A1, ETV6, CDK14
hsa04672	Intestinal immune network for IgA production	4	0.001522437	CCR10, TNFRSF13B, AICDA, HLA-DOB
hsa05340	Primary immunodeficiency	3	0.011947217	TNFRSF13B, AICDA, BLNK
hsa04640	Hematopoietic cell lineage	3	0.06510351	IL2RA, CD44, MME
hsa04064	NF-kappa B signaling pathway	3	0.067811577	CFLAR, BCL2A1, BLNK
hsa00533	Glycosaminoglycan biosynthesis - keratan sulfate	2	0.071387272	FUT8, CHST2

**Table 4:** Top 10 genes evaluated in the PPI network using five calculation methods (MCC, MNC, Degree, EPC, and EcCentricity) and employing CytoHubba in Cytoscape.

**gene_name**	**MCC**	**Gene name**	**MNC**	**gene name**	**Degree**	**gene name**	**EPC**	**gene name**	**EcCentricity**
**IRF4**	11	STAT3	6	IRF4	8	STAT3	10.782	STAT3	0.28571

**STAT3**	11	IRF4	5	STAT3	7	IRF4	10.659	IRF4	0.21429

**CD44**	8	CD44	5	CD44	5	CD44	10.346	CD44	0.21429

**MME**	6	MME	4	MME	4	IL2RA	9.487	IL2RA	0.21429

**ETV6**	4	ETV6	3	CCND2	4	MME	9.32	CCND2	0.21429

**LMO2**	4	IL2RA	3	CDK14	4	CCND2	9.148	CFLAR	0.21429

**CCND2**	4	CCND2	2	LMO2	4	CFLAR	8.598	BATF	0.21429

**CDK14**	4	CDK14	2	ETV6	3	BATF	8.404	NFKBIZ	0.21429

**IL2RA**	4	CFLAR	2	IL2RA	3	ETV6	8.374	MME	0.17143

**CFLAR**	3	HOPX	2	CFLAR	3	HOPX	7.399	ETV6	0.17143

**Table 5:** GO analysis and pathway enrichment for hub gene functions.

**Term**	**Description**	**Count**	**False discovery rate**	**Genes**
**Biological Process**				

**GO.0071345**	cellular response to cytokine stimulus	5	0.00579	CD44, IL2RA, IRF4, MME, STAT3

**GO.0014842**	regulation of satellite cell proliferation	2	0.0218	CFLAR, STAT3

**GO.0019221**	cytokine-mediated signaling pathway	4	0.0218	CD44, IL2RA, IRF4, STAT3

**Pathway**				

**4640**	Hematopoietic cell lineage	3	0.0011	CD44, IL2RA, MME

**4630**	JAK-STAT signaling pathway	3	0.00164	CCND2, IL2RA, STAT3

**5162**	Measles	3	0.00164	CCND2, IL2RA, STAT3

**5206**	MicroRNAs in cancer	3	0.00164	CCND2,CD44,STAT3

**4917**	Prolactin signaling pathway	2	0.0178	CCND2,STAT3

**4068**	FOXO signaling pathway	2	0.0447	CCND2,STAT3

**Table 6:** Enriched GO terms for the functions of the genes in the module.

**#Pathway ID**	**Pathway description**	**Observed gene count**	**False discovery rate**
GO.0050789	regulation of biological process	26	0.000803
GO.0048522	positive regulation of cellular process	25	2.51E-09
GO.0007165	signal transduction	25	9.18E-09
GO.0050794	regulation of cellular process	25	0.00158
GO.0044700	single organism signaling	24	1.99E-07
GO.0007154	cell communication	24	2.87E-07
GO.0051716	cellular response to stimulus	24	4.96E-06
GO.0010604	positive regulation of macromolecule metabolic process	22	2.02E-10
GO.0031325	positive regulation of cellular metabolic process	22	6.15E-10
GO.0050896	response to stimulus	22	0.00107
GO.0005829	cytosol	18	5.20E-05
GO.0005886	plasma membrane	17	0.0135
GO.0071944	cell periphery	17	0.0145
GO.0044459	plasma membrane part	13	0.00282
GO.0098552	side of membrane	11	2.81E-08
GO.0098589	membrane region	9	0.00338
GO.0045121	membrane raft	8	2.13E-06
GO.0009986	cell surface	8	0.00185
GO.0009897	external side of plasma membrane	7	2.37E-05
GO.1902494	catalytic complex	7	0.035
GO.0005515	protein binding	29	6.77E-12
GO.0005488	binding	28	0.0053
GO.0019899	enzyme binding	15	7.63E-07
GO.0005102	receptor binding	10	0.00191
GO.0060089	molecular transducer activity	10	0.0262
GO.0004672	protein kinase activity	7	0.00611
GO.0019904	protein domain specific binding	6	0.00546
GO.0019902	phosphatase binding	5	0.000899
GO.0019207	kinase regulator activity	5	0.00168
GO.0005126	cytokine receptor binding	5	0.00546

## Data Availability

The data used to support the findings of this study are available from the corresponding author upon request.

## Supplementary Materials

Supplementary Materials Figure S1: GO analysis classified the DEGs into three groups (molecular function, biological process, and cellular component). Figure S2: validation of hub gene expression in the Oncomine database. Figure S3: overall survival curve extracted from cBioportal related to the hub genes. Table S1: probable mutual exclusivity or cooccurrence of the hub genes.
