# Supplementary material for: Identification of Hub Genes and Key Pathways Associated with Two Subtypes of Diffuse Large B-Cell Lymphoma Based on Gene Expression Profiling via Integrated Bioinformatics
Source: Biomed Res Int. 2018 May 24;2018:3574534. doi: 10.1155/2018/3574534 (PMC5994323; doi:10.1155/2018/3574534)
Supplement: Supplementary Materials — Figure S1: GO analysis classified the DEGs into three groups (molecular function, biological process, and cellular component). Figure S2: validation of hub gene expression in the Oncomine database. Figure S3: overall survival curve extracted from cBioportal related to the hub genes. Table S1: probable mutual exclusivity or cooccurrence of the hub genes. [file 3574534.f1.zip › 3574534.f1/Table S1.docx]

| **Gene A** | **Gene B** | | **p-Value** | | **Association** | |  |
| --- | --- | --- | --- | --- | --- | --- | --- |
| MME | | CD44 | | 0.469678 | | Tendency towards mutual exclusivity | |
| MME | | IRF4 | | 0.599056 | | Tendency towards mutual exclusivity | |
| MME | | STAT3 | | 0.464043 | | Tendency towards mutual exclusivity | |
| MME | | IL2RA | | 0.312788 | | Tendency towards mutual exclusivity | |
| MME | | ETV6 | | 0.188738 | | Tendency towards co-occurrence | |
| MME | | CCND2 | | 0.615718 | | Tendency towards co-occurrence | |
| MME | | CFLAR | | 0.530322 | | Tendency towards co-occurrence | |
| CD44 | | IRF4 | | 0.530322 | | Tendency towards co-occurrence | |
| CD44 | | STAT3 | | 0.620644 | | Tendency towards co-occurrence | |
| CD44 | | IL2RA | | 0.575239 | | Tendency towards mutual exclusivity | |
| CD44 | | ETV6 | | 0.634238 | | Tendency towards mutual exclusivity | |
| CD44 | | CCND2 | | 0.634238 | | Tendency towards mutual exclusivity | |
| CD44 | | CFLAR | | 0.697662 | | Tendency towards mutual exclusivity | |
| IRF4 | | STAT3 | | 0.206738 | | Tendency towards co-occurrence | |
| IRF4 | | IL2RA | | 0.049721 | | Tendency towards co-occurrence (Significant) | |
| IRF4 | | ETV6 | | 0.615718 | | Tendency towards co-occurrence | |
| IRF4 | | CCND2 | | 0.188738 | | Tendency towards co-occurrence | |
| IRF4 | | CFLAR | | 0.469678 | | Tendency towards mutual exclusivity | |
| STAT3 | | IL2RA | | 0.095321 | | Tendency towards co-occurrence | |
| STAT3 | | ETV6 | | 0.275776 | | Tendency towards co-occurrence | |
| STAT3 | | CCND2 | | 0.054075 | | Tendency towards co-occurrence | |
| STAT3 | | CFLAR | | 0.379356 | | Tendency towards mutual exclusivity | |
| IL2RA | | ETV6 | | 0.496797 | | Tendency towards mutual exclusivity | |
| IL2RA | | CCND2 | | 0.503203 | | Tendency towards co-occurrence | |
| IL2RA | | CFLAR | | 0.424761 | | Tendency towards co-occurrence | |
| ETV6 | | CCND2 | | 0.000126 | | Tendency towards co-occurrence (Significant) | |
| ETV6 | | CFLAR | | 0.048643 | | Tendency towards co-occurrence (Significant) | |
| CCND2 | | CFLAR | | 0.365762 | | Tendency towards co-occurrence | |

**Table S1.** Probable mutual exclusivity or co-occurrence of the hub genes.
