# Supplementary figures and images for: Identification of Hub Genes and Key Pathways Associated with Two Subtypes of Diffuse Large B-Cell Lymphoma Based on Gene Expression Profiling via Integrated Bioinformatics
Source: Biomed Res Int. 2018 May 24;2018:3574534. doi: 10.1155/2018/3574534 (PMC5994323; doi:10.1155/2018/3574534)

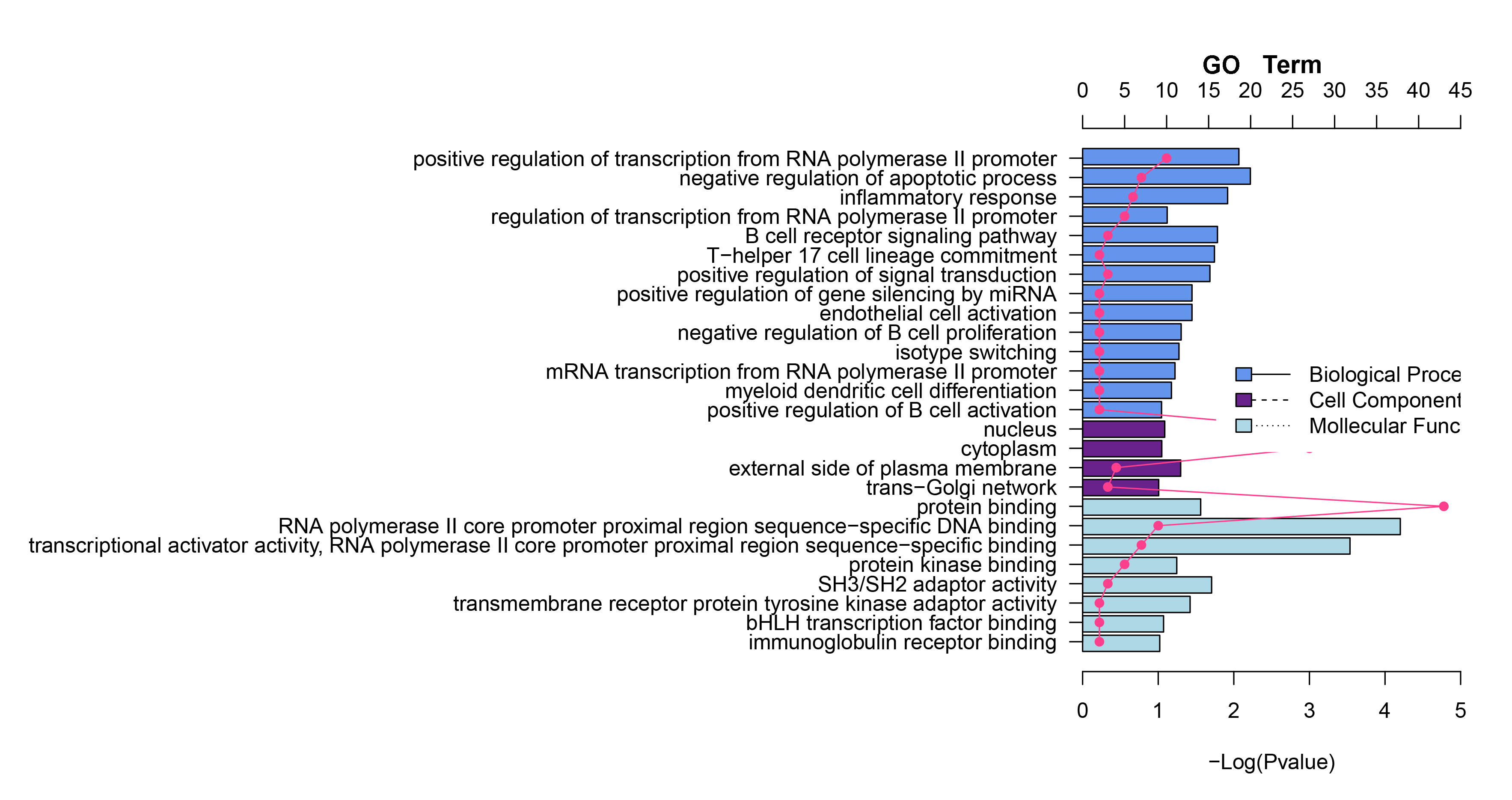

Supplement: Supplementary Materials — Figure S1: GO analysis classified the DEGs into three groups (molecular function, biological process, and cellular component). Figure S2: validation of hub gene expression in the Oncomine database. Figure S3: overall survival curve extracted from cBioportal related to the hub genes. Table S1: probable mutual exclusivity or cooccurrence of the hub genes. [file 3574534.f1.zip › 3574534.f1/S 1.jpg]

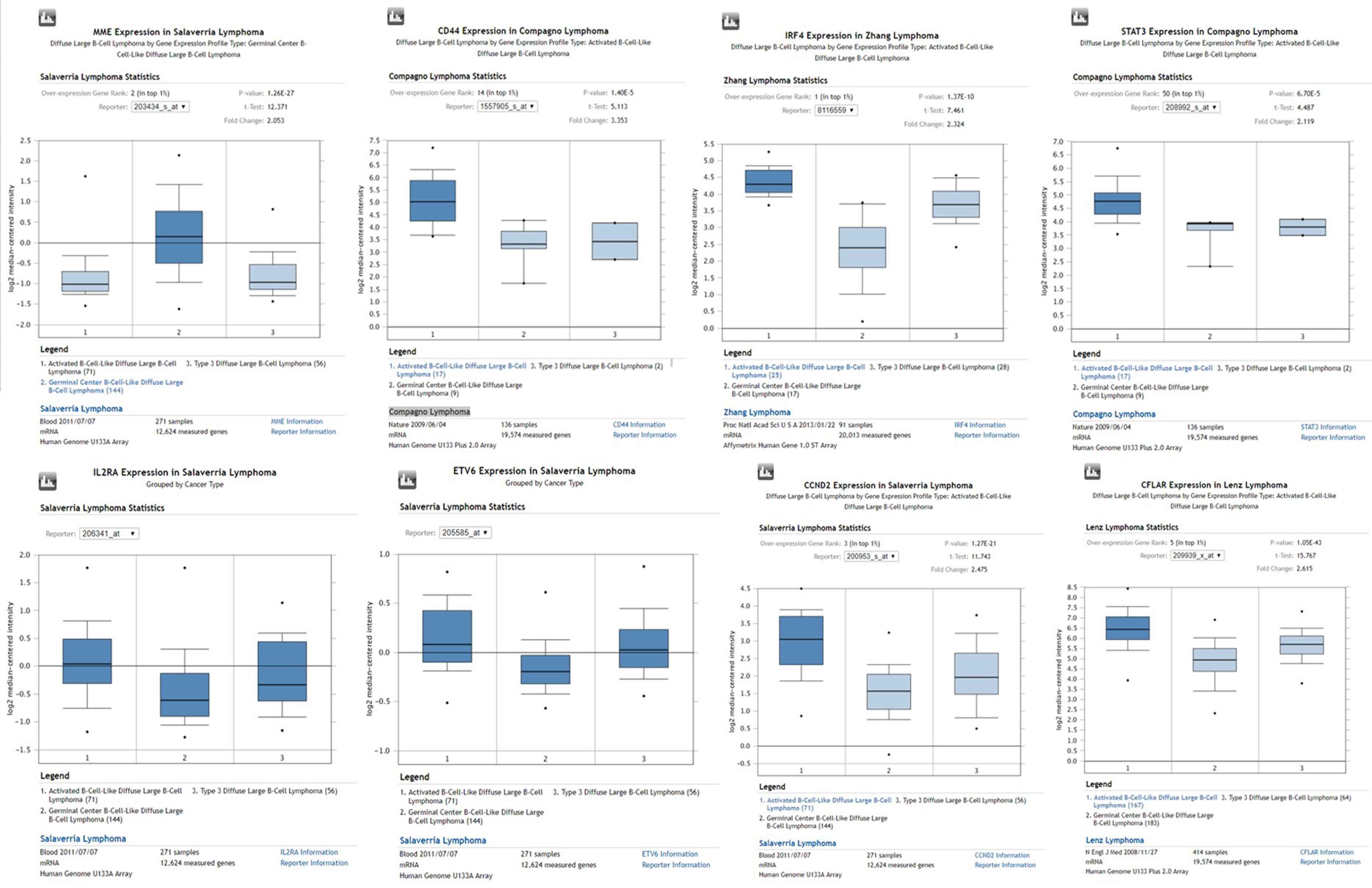

Supplement: Supplementary Materials — Figure S1: GO analysis classified the DEGs into three groups (molecular function, biological process, and cellular component). Figure S2: validation of hub gene expression in the Oncomine database. Figure S3: overall survival curve extracted from cBioportal related to the hub genes. Table S1: probable mutual exclusivity or cooccurrence of the hub genes. [file 3574534.f1.zip › 3574534.f1/S 2.jpg]

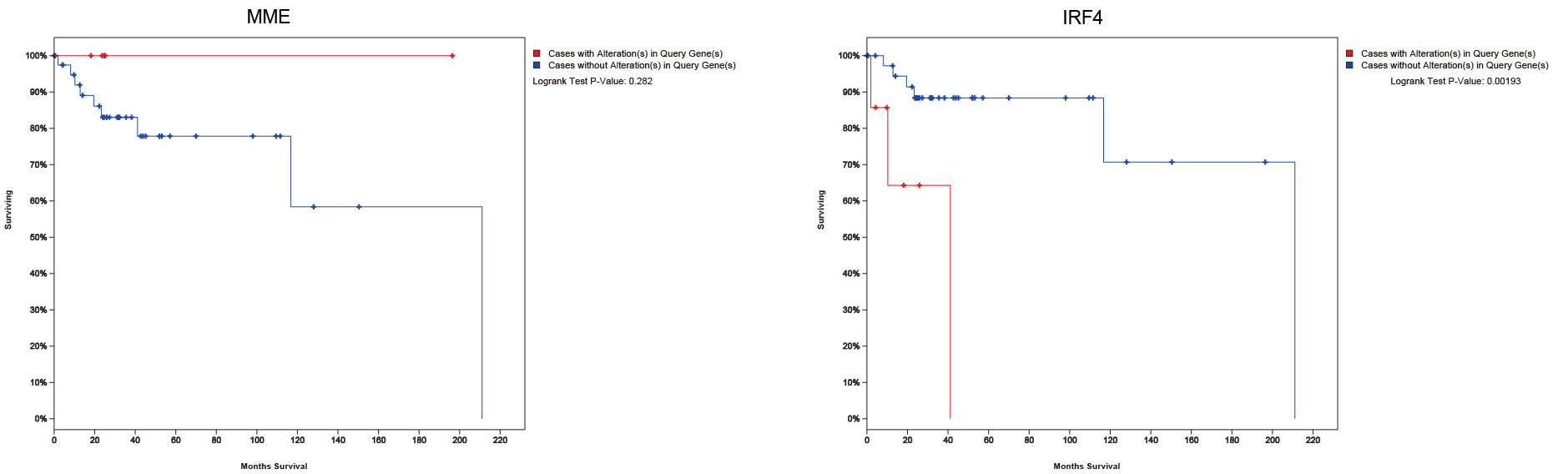

Supplement: Supplementary Materials — Figure S1: GO analysis classified the DEGs into three groups (molecular function, biological process, and cellular component). Figure S2: validation of hub gene expression in the Oncomine database. Figure S3: overall survival curve extracted from cBioportal related to the hub genes. Table S1: probable mutual exclusivity or cooccurrence of the hub genes. [file 3574534.f1.zip › 3574534.f1/S 3.jpg]
